# Pelvic metastatic squamous cell carcinoma of unknown primary site: A case report and brief literature review

**DOI:** 10.1097/MD.0000000000036796

**Published:** 2023-12-29

**Authors:** Qi-Zhou Zhu, Hui-Juan Li, Yuan-Qiang Li, Xiao-Hong Yu, Kuan-Yong Shu

**Affiliations:** a Department of Gynecological Oncology, Jiangxi Maternal and Child Health Hospital, Nanchang, Jiangxi, People’s Republic of China; b Medical Department, Jiangxi Maternal and Child Health Hospital, Nanchang, Jiangxi, People’s Republic of China; c Department of Pathology, Jiangxi Maternal and Child Health Hospital, Nanchang, Jiangxi, People’s Republic of China

**Keywords:** pelvic metastatic, squamous cell carcinoma, unknown primary site

## Abstract

**Rationale:**

Cancer with unknown primary site is a kind of disease that is difficult to deal with clinically, accounting for 2% to 9% of all newly diagnosed cancer cases. Here, we report such a case with pelvic metastatic squamous cell carcinoma of an unknown primary site and review the relevant literature.

**Patient concerns & Diagnoses:**

A 43-year-old Chinese female patient was referred to our hospital and initially diagnosed as “malignant tumor of right adnexal area?, obstruction of right ureter, secondary hydronephrosis”.

**Interventions:**

Thereafter cytoreductive surgery was performed which included a total hysterectomy, left adnexectomy, partial omentum resection, pelvic lymph node dissection, and para-aortic lymph node dissection. The primary lesion could not be identified by supplementary examination and postoperative pathology. The patient was diagnosed as pelvic metastatic squamous cell carcinoma whose primary site was unknown. To prevent a recurrence, we administered adjuvant chemotherapy for the patient.

**Outcomes:**

The patient was followed up after treatment, complete remission has been maintained for 72 months, and no recurrence or metastasis has been found.

**Lessons:**

Our case demonstrates that surgery combined with chemotherapy could be helpful for pelvic metastatic squamous cell carcinoma of unknown primary site.

## 1. Introduction

Cancer with unknown primary site (CUP) is a kind of disease that is difficult to deal with clinically and accounts for 2% to 9% of all new cancer cases.^[1]^ Such a group of heterogeneous tumors primary location cannot be determined. Studies suggest that their common clinical features may be early spreading and irregular patterns of metastasis.^[2,3]^ More than 60% of the pathological types were adenocarcinoma, just only 5% were squamous cell carcinoma.^[1,4]^ There is no established model of a treatment plan, and the individual differences are huge. This study reported a case of “adnexal malignant tumor” which was found to be pelvic metastatic squamous cell carcinoma whose primary lesion was unknown after surgery, and the related literature was reviewed for guiding clinical practice.

## 2. Case description

A 43-year-old Chinese female patient was referred to our hospital on March 15, 2017, with right lumbosacral pain for more than 1 month. Gynecological examination: no abnormality of vulva and vagina; cervical diameter was 2.5 cm, and cervix had a little erosion; The body of uterus was anteversion, 5 cm × 4 cm × 3 cm in size; A mass of about 6.0 cm in diameter was palpable in the right adnexal area, and the boundary with the surrounding area was unclear. Vagino-recto-abdominal examination: no nodule was found in the rectum fossa, an induration of about 2.5 cm in diameter was palpable on the right pelvic wall, which is fixed and immobile.

**Figure 1.**
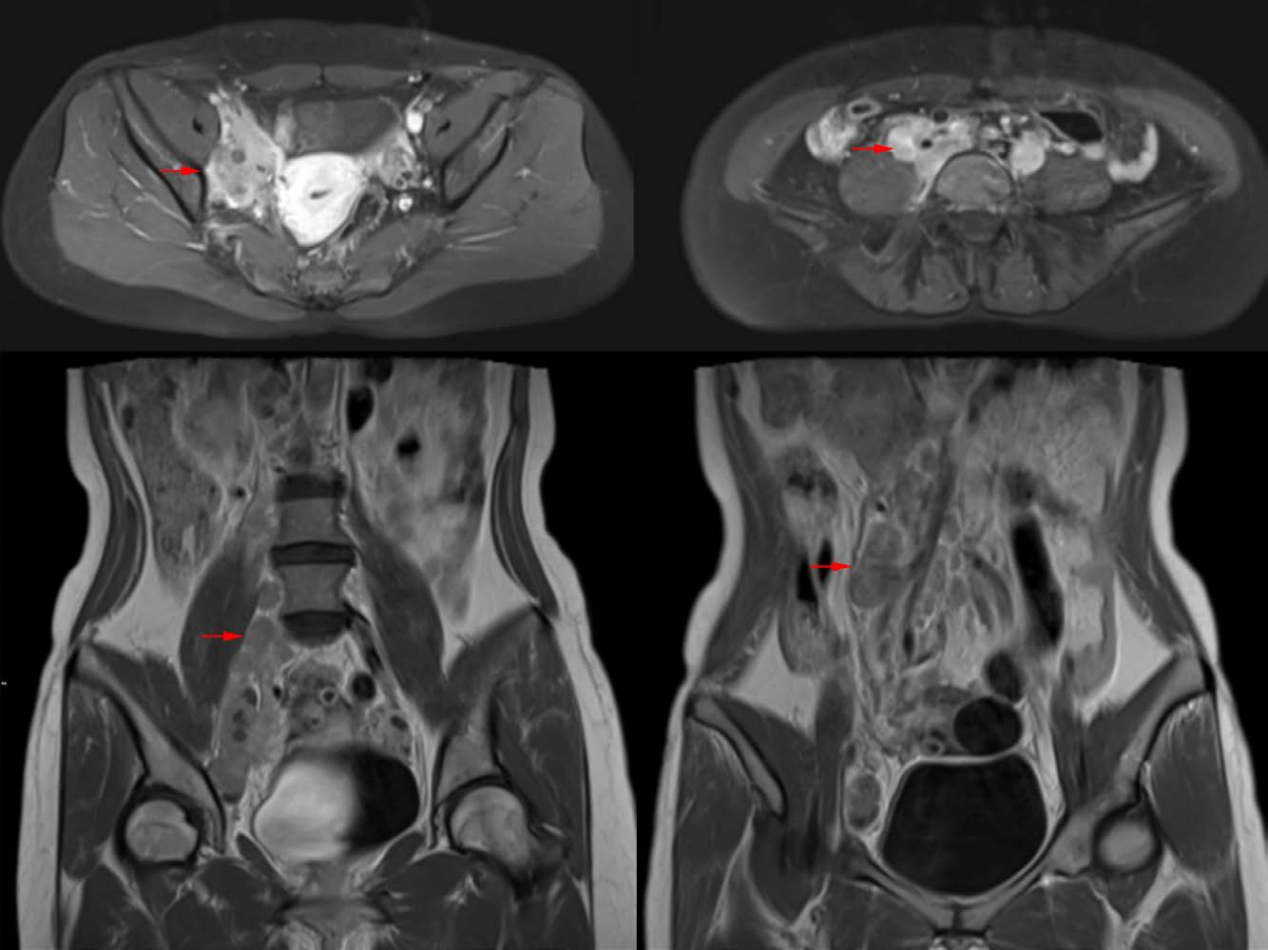
Magnetic resonance imaging (MRI) with contrast findings.

Magnetic resonance imaging (MRI) with contrast showed: cystic - solid mass in front of the uterus, considered as a malignant tumor of right adnexal origin (7.05 cm × 4.03 mm × 3.72 cm, the internal signal was uneven, showing long T1, slightly long T2 and long T2 signals. Diffusion weighted imaging showed solid components were significantly high signal, and enhanced scan showed solid components were significantly enhanced); the unclear boundary between mass and ureter, indicating ureteral maybe invasion with right ureteral dilatation; multiple lymph nodes in retroperitoneum and right iliac vessels (maximum 1.82 cm × 4.14 cm), considered as metastasis (shown in Fig. 1). Abdominal computed tomography (CT) showed right side hydronephrosis and decreased renal perfusion. Chest CT showed that the small nodules in the lower lobe of the left lung were supposed to be proliferative foci, scattered calcified foci in both lungs, and localized thickening of bilateral pleura; no special lesions were found under gastroscopy and colonoscopy (In order to exclude the possibility of gastrointestinal metastasis). Tumor markers: CA125: 72.39 u/mL, HE4: 134.1 pmol/L, SCC: 56.08 ng/mL, CEA: 81.35 ng/mL.

**Figure 2.**
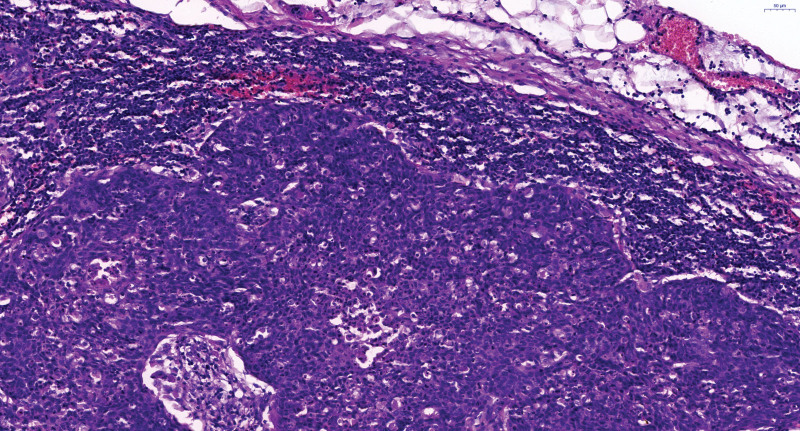
Postoperative pathological findings, HE × 200.

**Figure 3.**
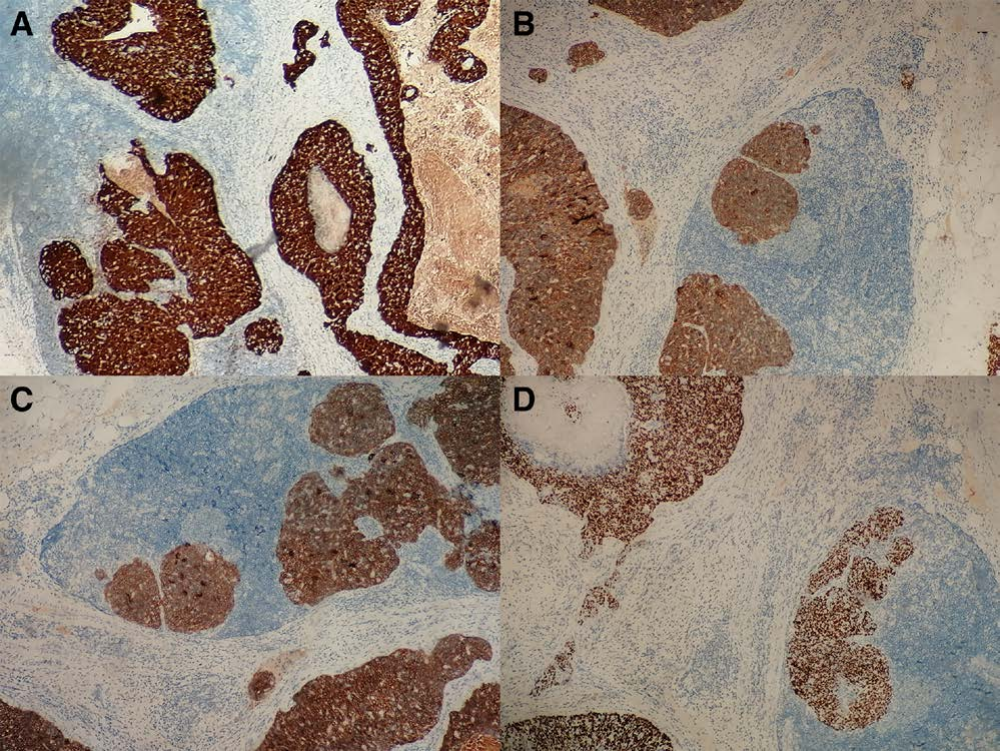
Immunohistochemistry staining tests findings: (A) p16 (+ +), (B) P40 (+ +), (C) ck10/13 (+ +), (D) p63 (+), IHC × 200.

An exploratory laparotomy was performed on March 23rd, 2017. During the operation, the polycystic right ovary was found to be 7.0 cm × 4.5 cm × 4.5 cm in size. Edema on the right side of the fallopian tube and some miliary nodules were scattered in it. The fusion of the right lower part of the ureter with pelvic metastatic lymph nodes about 3.0 cm at length, and the upper ureter showed dilatation and hydronephrosis. The lymph nodes of the right pelvic wall, right presacral, and right abdominal aorta were fused in mass, extending to the level of renal vein, with the largest of 5.0 cm × 3.5 cm × 3.0 cm. The right adnexa and the right common iliac lymph node were resected and frozen pathological sections showed: The stroma of ovarian tissue was loose and edematous, and its internal vessels were proliferous with cyst formation; Right common iliac lymph node carcinoma. Considering the possibility of peritoneal cancer and communicating with the patient’s family members, cytoreductive surgery was then performed which included a total hysterectomy and left adnexectomy, partial omentum resection, pelvic lymph node dissection, para-aortic lymph node dissection, and bilateral ureteral stent placement under cystoscope. Right ureter was successfully dissected from the mass. Postoperative pathology indicated: metastatic carcinoma of lymph nodes in right ilium (2/2), right external iliac (5/5), right inguinal depth (6/6), right obturator (3/3 + 2 cancer nodules), right presacral (7/7), para-aortic (9/10), right common iliac (6/6) lymph node metastasis. Combined with HE and IHC, all of them were consistent with squamous cell carcinoma. Cancer foci were found in the right round ligament, right pelvic wall peritoneum, right mesosalpinx, and fibrovascular adipose tissue around the left fallopian tube while no cancer was found in the uterus, ovaries, right fallopian tube, and other lymph nodes. Immunohistochemistry staining tests included: p16 (+ +), P40 (+ +), ck10/ 13 (+ +), AE1/ AE3 (+), p63 (+), CK high (+), EMA part (+), CEA (+), CK5/ 6 (+), er (−), PR (−), p53 (−), VIM (−), CA125 (−), CK7 (−), CK20 (−), WT-1 (−), Pax-8 (−), Cdx-2 (−), villin (−), LCA (−), CK (low −), CK8/ 18 (−), Ki67 (about 80%) (shown in Fig. 2 and Fig. 3).

We tried to find the primary lesion; Positron emission tomography-computed tomography (PET-CT) was performed in April 2017. The results showed: the gastric wall of the curvatura ventriculi major of the stomach was slightly thickened, and the metabolism was increased; there were multiple nodular soft tissue shadows in the retroperitoneum (right renal hilum and left L5 vertebral body) and left supraclavicular fossa with a high metabolism, which indicates metastatic lymph nodes; right hydronephrosis, right ureter dilatation; Calcification in the upper lobe of the left lung. Unfortunately, the primary lesion could not be identified according to the results of PET-CT. To prevent a recurrence, after doctor - patient communication, adjuvant chemotherapy was administered: paclitaxel liposome (175 mg/ m^2^ ivgtt D1) and nedaplatin (80–100 mg/m^2^ ivgtt D2), which lasts for 8 cycles and interval of 21 days.

The patient was discharged from the hospital in September 2017 and followed up regularly. In the fifth month after discharge, serum SCC-Ag increased (21.82 ng/mL), but no new lesions were found in systemic imaging examination. One month later, serum SCC-Ag returned to normal (0.51 ng/mL). Up to date, complete remission has been maintained for 72 months. There was no evidence of recurrence in tumor markers, B-ultrasound, MRI, or CT.

## 3. Discussion

Cancers of unknown primary (CUP) is defined as a metastatic malignant tumor confirmed by histopathology, but its primary sites cannot be identified in the initial treatment and further evaluation.^[5,6]^ The clinical characteristics of heterogeneous tumors in this group varied and the prognosis was poor. Their common features are metastasis early, strong invasion, and unpredictable metastasis mode. The overall median survival time is only 8 to 12 months.^[3,5]^ The incidence of CUP in men and women was roughly the same, with an average age of 60 at diagnosis. In 2020, it is estimated that 30,270 cases of CUP will be diagnosed in the United States, accounting for about 2 % of all new cancers.^[7]^ There are little relevant literature and a lack of Big Data statistics in China, so it is impossible to know the systematic data of CUP in China.

Multiple metastases were observed in more than 50% of patients with CUP, and the common sites of involvement were liver, lung, bone, and lymph nodes.^[8,9]^ Although some patterns of metastasis may indicate a possible primary site, doctors should not rely on the known patterns of metastatic tumors to determine the primary site, since CUP can metastasize to any location. Most of the patients with CUP had a poor prognosis, and the risk factors included male genital system, multiple organ metastasis (such as liver, lung, bone), non-papillary malignant ascites (adenocarcinoma), peritoneal metastasis, multiple brain metastasis, multiple lung/pleura adenocarcinoma and bone lesion formation.^[10,11]^ In this case, ovarian cancer or fallopian tube cancer with pelvic lymph node metastasis was considered most likely before the operation. But the final results showed that no tumor was found in the uterus and adnexa. Metastatic cancer was concentrated in lymph nodes and surrounding connective tissue of pelvic organs. Neither preoperative assessment nor postoperative evaluation succeeded to identify the primary lesion. Therefore, the postoperative diagnosis of this patient was still unclear. It was considered as CUP, suggesting that we should consider various possibilities in clinical practice for patients with pelvic tumors. We should collect relevant information about the primary site of the tumor before the operation as much as possible to facilitate treatment.

Squamous cell carcinoma can metastasize to the head and neck, supraclavicular, axillary, and inguinal lymph nodes. It is recommended that patients with inguinal lymph node squamous cell carcinoma should undergo colonoscopy, enhanced CT scan of the chest, abdomen, and pelvis.^[12]^ For patients with pain, a bone scan (if chest/abdomen/pelvis contrast-enhanced CT scan has not been performed previously) and diagnostic imaging studies are recommended. CT, MRI, gastrointestinal endoscopy, and PET-CT have been performed in this patient, but no further diagnosis was made. At the same time, we should consider other technical means, such as bone scan or PET-MRI.^[13]^ On the 1 hand, gene expression profile and molecular cancer classification could be used to determine the origin of the tumor, to guide the treatment of specific sites. On the other hand, next-generation sequencing could be used to identify genomic aberrations that can be targeted for treatment.^[14,15]^ In consideration of all aspects of the results, so we can formulate a reasonable treatment plan.

Combined with the existing studies, lymph node dissection is recommended for patients with site-specific squamous cell carcinoma, and local axillary or inguinal lymph node involvement. If the lesion is limited, radiotherapy combine with chemotherapy can be considered. If there is chemotherapy contraindication or chemotherapy cannot be tolerated, radiotherapy alone can be considered.^[16]^ Platinum-based chemotherapy regimens are usually more effective.^[17]^ Historically, cisplatin combined with 5-FU is the most commonly used treatment for patients with unknown primary squamous cell carcinoma.^[18]^ At present, paclitaxel/carboplatin or cisplatin, cisplatin/gemcitabine, and docetaxel/cisplatin or carboplatin are more commonly used.^[19]^ Recently, anti-PD-1 antibody (pembrolizumab) has been reported in patients with unresectable or metastatic microsatellite instability-high/different mismatch repair solid tumors.^[20]^ These patients always relapse and have no satisfactory alternative treatment, but CUP patients are generally considered to have a lower risk of microsatellite instability-high/different mismatch repair.^[15]^ In this case, the pelvic lymph nodes and connective tissue around the organs had extensive metastasis. Cytoreductive surgery reached the level R0. The extent of the operation was reasonable. Postoperative PET-CT indicated multiple nodular soft tissue shadows in the left supraclavicular fossa with increased metabolism, which was considered metastatic lymph nodes. Considering the limited effect of pelvic local radiotherapy, Paclitaxel liposomes and nedaplatin were used for chemotherapy lasts for 8 cycles after the operation. The patient’s serum tumor markers gradually decreased to normal, and the right ureteral dilatation and hydronephrosis were significantly alleviated. The patients were followed up regularly afterward. Although there was a transient increase of serum SCC-Ag after discharge, there was no evidence of imaging recurrence. At present, the patients were followed up for 72 months and still in a state of complete remission. It shows that the curative effect of the patient after the operation, chemotherapy, and other comprehensive treatment is obvious, and the next step is to continue to closely follow-up with the patient. Our case demonstrates that surgery combined with chemotherapy may be helpful for pelvic metastatic squamous cell carcinoma of unknown primary site.

## 4. Conclusion

In summary, the clinical symptoms of pelvic metastatic tumors with the unknown primary site are diverse due to individual differences. The common characteristics of these tumors are early metastasis, strong invasion, and unpredictable metastasis mode. Therefore, we should collect as much information about patients as possible before surgery. If available, PET-CT/MRI, bone scan, gene test, and other examinations should be carried out to further prompt the diagnosis. Comprehensive treatments such as surgery, chemotherapy, radiotherapy, or targeted therapy should be actively adopted. It is believed that with the accumulation of experience, we would have more available diagnostic methods and treatment measures for this kind of rare diseases.

## Acknowledgments

This work was supported by the Leading Discipline of Medicine in Jiangxi Province and Jiangxi Gynecological Cancer Medical Center.

## Author contributions

**Conceptualization:** Qizhou Zhu, Kuan-Yong Shu

**Data curation:** Qizhou Zhu, Hui-Juan Li, Yuan-Qiang Li

**Formal analysis:** Qizhou Zhu, Hui-Juan Li

**Funding acquisition:** Qizhou Zhu

**Investigation:** Qizhou Zhu, Hui-Juan Li, Yuan-Qiang Li, Xiao-Hong Yu

**Methodology:** Qizhou Zhu, Xiao-Hong Yu

**Project administration:** Qizhou Zhu

**Supervision:** Kuan-Yong Shu

**Writing – original draft:** Qizhou Zhu, Yuan-Qiang Li

**Writing – review & editing:** Qizhou Zhu
